# Psoriasis Course in Patients with Alopecia Areata Undergoing Baricitinib Therapy

**DOI:** 10.3390/clinpract16020028

**Published:** 2026-01-28

**Authors:** Enrico Matteini, Fabio Artosi, Giacomo Caldarola, Lorenzo Maria Pinto, Alfredo Rossi, Lorenzo Ala, Gaetana Costanza, Luca Bianchi, Elena Campione, Laura Diluvio

**Affiliations:** 1 Dermatology Unit, Department of Systems Medicine, University of Rome “Tor Vergata”, 00134 Rome, Italy; ematteini13@gmail.com (E.M.); fabio.artosi994@gmail.com (F.A.); luca.bianchi@uniroma2.it (L.B.); campioneelena@gmail.com (E.C.); lauradiluvio@yahoo.it (L.D.); 2 UOC di Dermatologia, Dipartimento di Scienze Mediche e Chirurgiche, Fondazione Policlinico Universitario “A. Gemelli”—IRCCS, 00168 Rome, Italy; giacomo.caldarola@policlinicogemelli.it (G.C.); lorenzomaria.pinto@gmail.com (L.M.P.); 3 UOC di Dermatologia, “Sapienza” University of Rome, 00185 Rome, Italy; alfredo.rossi@uniroma1.it (A.R.); lorenzoala@hotmail.it (L.A.); 4 Department of Experimental Medicine, University of Rome “Tor Vergata”, 00134 Rome, Italy

**Keywords:** alopecia areata, baricitinib, psoriasis, psoriatic arthritis, JAKi

## Abstract

**Background/Objectives:** Alopecia areata (AA) and psoriasis are immune-mediated diseases that can coexist, suggesting shared pathogenic mechanisms. While Janus kinase inhibitors (JAKi) are approved for AA treatment, their role in managing concomitant psoriasis and psoriatic arthritis (PsA) remains unclear. This study evaluates the efficacy and safety of baricitinib in patients with severe AA and coexisting psoriasis and/or PsA. **Materials and Methods:** A retrospective case series of five patients (mean age 53.2 years) with severe AA (SALT > 80) or alopecia universalis (AU) and concomitant psoriasis (*n* = 2) and/or PsA (*n* = 3) was conducted in the Dermatology Unit of Policlinico of Tor Vergata, Catholic University of the Sacred Heart and La Sapienza University of Rome, Italy. Patients received baricitinib 4 mg/day and were assessed at weeks 4, 24, and 52 using SALT, PASI, and pVAS scores. **Results:** At week 52, one patient achieved complete AA remission, while two improved to SALT < 20 (mean SALT 83 to 8.75). Psoriasis remained stable (mean PASI 1.4 to 0.5). However, one PsA patient worsened (pVAS 9) and discontinued the treatment. **Conclusions:** Baricitinib was effective for AA, with potential benefits for psoriasis, but it may not be optimal for PsA. Further studies are needed to define its role in multiple immune diseases.

## 1. Introduction

Alopecia areata (AA) is an inflammatory, non-cicatricial type of hair loss disorder, with a lifetime risk of approximately 2% worldwide. AA is considered the most prevalent autoimmune disorder and the second most prevalent hair loss disorder [1]. In 75% of patients, the disease is limited to the scalp, even though it can affect every part of the body. Typical lesions usually appear as single or multiple patches, though specific clinical patterns can be found, such as ophiasis, sisaipho, total scalp hair loss, alopecia totalis and alopecia universalis [2]. AA is a chronic relapsing condition that can have considerable effects on the patients’ quality of life, correlated with the severity of the disease [3].

Psoriasis is a long-term recurrent systemic inflammatory condition affecting the skin and/or joints, with a reported prevalence ranging from 0.09% to 11.4% [4]. Plaque psoriasis is the most common variant of the disease. Dry and raised plaques and silvery scales on the skin are the most common signs, while synovitis, enthesitis and marginal bone erosions of the peripheral and axial skeleton are the typical joint signs [5]. Other clinical variants include guttate psoriasis, typically occurring in younger individuals and frequently triggered by bacterial infections, and characterized by small, erythematous, scaly, oval-shaped macules predominantly involving the trunk and extremities. Inverse psoriasis primarily affects intertriginous sites, while pustular psoriasis may present in localized forms (such as palmoplantar) or as generalized and severe disease. Erythrodermic psoriasis represents a rare but potentially life-threatening variant.

Refs. [6,7] indicate that, given the significantly debilitating nature resulting from joint involvement, especially if left untreated, and the considerable aesthetic impact that particularly severe cutaneous forms can cause, psoriasis can often lead to a significant deterioration in the quality of life of affected patients [8].

Psoriasis can be associated with various comorbidities, including metabolic disorders, an increased risk of cardiovascular diseases, anxiety and/or depression, and other autoimmune conditions, such as AA, a chronic autoimmune and inflammatory disorder at the hair follicle level [9].

As reported in a recent study, the pooled prevalence of AA among patients with psoriasis was 0.5%, whereas the pooled prevalence of psoriasis among patients with AA was 2.5%. Jung and colleagues considered the degree of bidirectional association in younger participants to be “noteworthy” and found that patients with psoriasis, regardless of their age, had significantly higher odds for AA and vice versa [9].

The causal link between these two pathologies does not seem to be fully known, but there are definitely some clinical affinities that may even cause a form of non-scarring cicatricial alopecia with preservation of hair follicle units. Recent articles on patients with concurrent psoriasis and AA describe normal hair regrowth in areas of skin with plaque psoriasis, offering information on the immune factors underlying each disease [10]. Furthermore, many of the same therapies, e.g., topical or intralesional, or systemic steroids, are used to treat both diseases; this emphasizes both their overlapping characteristics and the lack of targeted therapy.

Since traditional therapies are not sufficiently effective and are burdened by serious side effects, new therapeutic modalities must be explored. Janus kinase inhibitors (JAKi) represent a new class of medications that act by selectively or non-selectively inhibiting the Janus kinase (JAK)—signal transducer and activator of transcription (STAT) signaling pathway. The Janus kinase/signal transducer and activator of transcription (JAK/STAT) pathway plays a central role in the pathogenesis of several immune-mediated dermatologic diseases, including AA, psoriasis, and atopic dermatitis (AD). In AA, dysregulated IFN-γ and IL-15 signaling via JAK1 and JAK2 promotes cytotoxic CD8+ T cell-mediated follicular damage [11]. Although psoriasis is primarily driven by the IL-23/Th17 axis, JAK-dependent signaling of cytokines such as IL-6 and IFN-γ also contributes to keratinocyte hyperproliferation and chronic inflammation [12]. In AD, Th2 cytokines, including IL-4 and IL-13, engage JAK1 and JAK3, perpetuating inflammation and barrier dysfunction [13]. The shared involvement of JAK signaling across these conditions provides the molecular basis for the clinical efficacy of JAK inhibitors, such as baricitinib, in patients with overlapping AA and psoriasis. Baricitinib is a selective and reversible inhibitor of JAK1/JAK2, already used as a therapy for rheumatoid arthritis/juvenile idiopathic arthritis and AD from the age of 2, and recently approved by the FDA for the treatment of moderate-to-severe AA in adult patients, with satisfactory results reported in the literature [14,15].

The JAK-STAT signaling pathway regulates the activity of numerous cytokines, including interleukins (IL)-17 and -23, which play a central role in the pathogenesis of psoriasis [16,17], thus JAKi could also represent a valid therapeutic alternative for psoriasis. Currently, two JAKi are approved for psoriasis: Upadacitinib, a selective JAK1 inhibitor, indicated for the treatment of Psoriatic arthritis (PsA), which modulates signaling downstream of pro-inflammatory cytokines such as IL-6, IFN-γ, and IL-23, thereby dampen both innate and adaptive immune responses involved in joint inflammation and synovial hyperplasia [18], and Deucravacitinib, a highly selective allosteric inhibitor of TYK2, indicated for moderate-to-severe plaque psoriasis affecting the skin only; unlike classical JAK inhibitors binding the ATP-binding site, deucravacitinib targets the regulatory pseudokinase domain of TYK2, resulting in a more specific inhibition of IL-23, IL-12, and type I IFN signaling [19,20]. However, cases of paradoxical psoriasis induced by baricitinib have been reported in the literature; therefore, it is necessary to gain more experience in the use of these drugs in plaque psoriasis [21].

We report our experience with patients affected by severe AA and concomitant psoriasis and/or psoriatic arthritis, treated with baricitinib 4 mg/day.

## 2. Materials and Methods

We enrolled 5 patients (2 males, 3 females; mean age 53.2 years, with a minimum age of 27 years and a maximum age of 63 years) from the Dermatology Unit of Policlinico of Tor Vergata, Catholic University of the Sacred Heart and La Sapienza University of Rome, Italy. All patients were affected by a severe form of AA with concomitant cutaneous and/or articular psoriasis (1 patient with plaque psoriasis, 1 patient with guttate psoriasis, 3 patients with psoriatic arthritis, PsA). Reported comorbidities included arterial hypertension, type II diabetes mellitus, and hypothyroidism (Table 1). The comorbidities observed in our cohort did not change during the observation period, nor did they influence the disease course or treatment adherence. The study was conducted in accordance with the Declaration of Helsinki, and the consent of the local ethical committee was not required in Italy for the real-life clinical study.

All patients had previously received conventional therapies for AA, including topical and/or systemic corticosteroids and cyclosporine, without significant benefits. Among them, the three patients with PsA had undergone treatment with conventional synthetic and biological Disease-Modifying Anti-Rheumatic Drugs (csDMARDs and bDMARDs), specifically Ixekizumab (two patients) and Brodalumab (one patient). While these treatments provided partial or complete control of joint symptoms, they had a minimal impact on AA. The two patients with only cutaneous psoriasis had been managed with cycles of local corticosteroid therapy, achieving satisfactory disease control.

AA assessment was performed through clinical evaluation, using the Severity of Alopecia Tool (SALT) score, and dermoscopy, with the Dermaview digital dermoscope and DL200 Hybrid dermoscope (Dermlite, San Juan Capistrano, CA, USA), at a magnification of 10×. The SALT score describes the percentage of scalp hair loss and ranges from 0 to 100. A SALT score of 100 means complete (or 100%) scalp hair loss, while limited (mild) hair loss represents 1–20% scalp involvement, moderate hair loss represents 21–49% scalp involvement and severe hair loss represents 50–100% scalp involvement [22].

Psoriasis assessment was performed using the Psoriasis Area Severity Index (PASI), ranging from 0 (no psoriasis) to 72 (extremely severe psoriasis), for the cutaneous component and the Pain Visual Analogue Scale (pVAS), patient-reported pain level, measured on a visual analogue scale ranging from 0 to 10 cm for the articular component.

The three patients previously receiving biological therapy discontinued their treatment and completed an appropriate washout period before initiating baricitinib. Following instrumental and hematological screening, baricitinib was administered at a dose of 4 mg/day. Patients were evaluated at baseline and subsequently at 1 month (Week 4), 6 months (Week 24), and 12 months (Week 52) of therapy.

## 3. Results

In our case series, two patients were affected by a severe form of AA with a SALT score >80, and 3 patients with a form of AU (SALT score > 100). At baseline, the mean SALT (mSALT) score was 83, while a good control of the psoriatic disease was observed, with a mean PASI (mPASI) of 1.4 and a mean pVAS of 0.

At Week 4, 2 patients exhibited partial clinical remission of AA, while in 3 patients, clinical stability was observed with a marked improvement on trichoscopic evaluation, with vellus hair and other signs of regrowth in the affected areas. The mSALT after one month of treatment was 57. No significant changes were observed in cutaneous or articular psoriasis.

**Figure 1:**
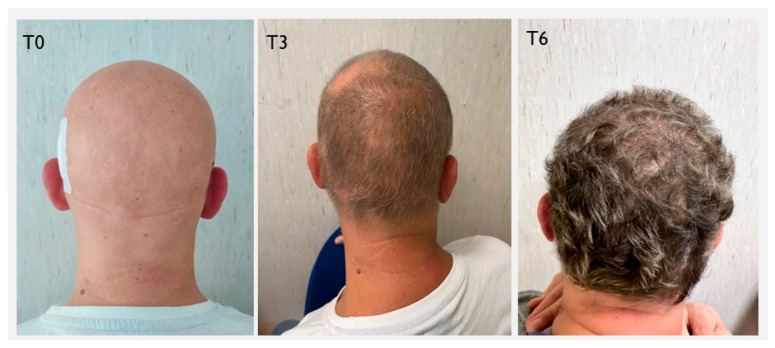
Evolution of hair growth at baseline (T0) and following treatment with baricitinib (T3 and T6) in a patient affected by psoriasis.

**Figure 2:**
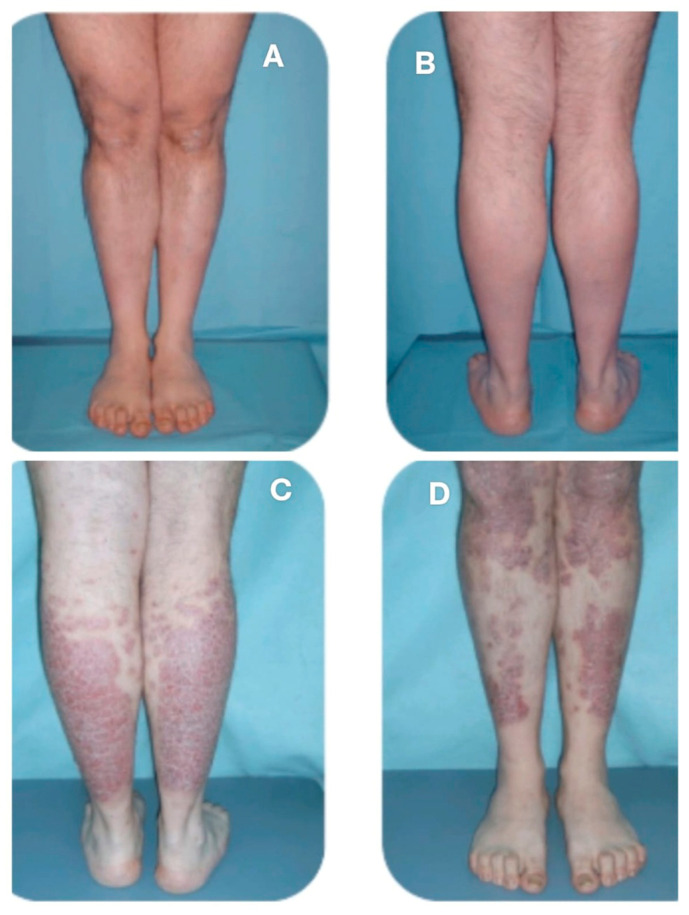
(**A**,**B**): Clinical appearance of the lower limbs prior to baricitinib initiation, in a patient previously treated with anti-IL-17 biologic therapy. (**C**,**D**): Clinical appearance of the lower limbs after discontinuation of anti-IL-17 therapy and 52 weeks of baricitinib treatment. This case represents the only example in our case series in which switching from a conventional bDMARD to the JAK inhibitor baricitinib failed to maintain adequate long-term control of psoriatic disease, both cutaneous and articular. While the patient had previously achieved good disease control on biologic therapy, the transition to baricitinib—despite yielding significant improvement in AA—was associated with a progressive relapse of both skin and joint symptoms over time.

At Week 24, one of the patients with PsA spontaneously decided to discontinue therapy due to the lack of evident clinical results on AA, despite improvements observed on trichoscopy and no cutaneous or articular exacerbation of the psoriatic condition. In the remaining 4 patients, clinical improvement of AA was observed (mSALT 22.5), with one of the AU group patients achieving a complete clinical remission (SALT 0) (Figure 1). The psoriasis condition remained stable. At Week 52, one patient achieved complete clinical remission of AA, while the other two improved to a mild stage of the condition, with a SALT score < 20. The mSALT score was 8.75. Clinical improvement was also noted in cutaneous psoriasis, with a mPASI score of 0.5. None of the three patients reported joint pain. However, the fourth patient, affected by PsA, despite achieving complete clinical remission of AA (SALT 0 since Week 24), experienced significant worsening of joint disease, with a patient-reported pVAS score of 9, and a flare of the cutaneous manifestations, predominantly affecting the lower limbs, with a PASI score of 12 (Figure 2A–D), and nail involvement. This patient subsequently discontinued baricitinib therapy and transitioned to alternative treatments.

## 4. Discussion

Together with previous studies on the constellation of autoimmune disorders, our case series presented the coexistence of psoriasis and AA in the same patient, suggesting shared pathogenetic mechanisms. Both AA and psoriasis are considered T-cell-mediated dermatoses. Among the various T-cell subsets associated with the diseases, T-helper (Th)1 and Th17 can be linked with AA and psoriasis [23]. As demonstrated by Suarez Farinas et al., in comparative transcriptome analysis using lesional skins of patients with AA and psoriasis and non-lesional control skins, Th1/interferon-related markers were increased in both diseases [8]. Moreover, IL-17, mainly produced by Th17, was reportedly involved in the pathogenesis of both psoriasis and AA [24].

Furthermore, systemic treatments with tumor necrosis factor-α inhibitors, cyclosporine, phosphodiesterase-4 inhibitors, and other biologics targeting IL-12/23 or IL-17 for psoriasis were also reported as involved in the development of AA [9].

In the literature, there are case reports of patients with AA treated with JAKi approved for certain forms of arthritis. Todberg et al. described a complex case of a male patient affected by psoriasis, PsA, and AA, who achieved a significant improvement in both rheumatological and dermatological conditions with tofacitinib [25]. More recently, in 2023, Kolcz et al. reported a case involving a 14-year-old adolescent with AD and AU successfully treated with upadacitinib, a JAKi already approved for PsA treatment [26]. In contrast, apart from an RCT investigating its use in juvenile idiopathic arthritis, there are no studies in the literature describing the use of baricitinib in other forms of arthritis [27].

Baricitinib has been widely used in dermatology as a new molecular-targeted therapy. Increasing evidence suggests that baricitinib is effective against AD, AA, psoriasis, and vitiligo. Many inflammatory dermatoses are driven by inflammatory mediators that rely on JAK/STAT signals, and the use of JAK inhibitors has become a new strategy for the treatment of diseases for which conventional drugs have not been effective [28].

Our experience has highlighted that baricitinib, the first JAKi approved for AA, is not indicated for psoriasis, but could represent a valid alternative therapeutic treatment in patients affected by both conditions. In our sample, 3 out of 5 patients had already undergone biological therapies with anti-TNF-alpha, anti-IL-17, and anti-IL-23, often discontinued despite good control over psoriasis due to the lack of efficacy, or even worsening, on AA. Our data not only suggests, as indicated in the literature, the efficacy of baricitinib on alopecia, but also a potential efficacy in controlling psoriatic pathology, whilst maintaining joint stability and improving PASI. Only one patient experienced PsA worsening following the switch from bDMARD (Ixekizumab) to Baricitinib, thus indicating the need to further investigate the efficacy of this drug on the joint component. This case illustrates the complexity of therapeutic decision-making in patients with coexisting severe AA and PsA. The patient chose to discontinue a previously effective bDMARD regimen—under which both cutaneous and joint manifestations were well controlled—in favor of a therapy specifically targeting alopecia, driven by the substantial psychological burden it imposed. This decision, however, resulted in a loss of overall disease control, affecting not only the skin, which is primarily an aesthetic concern, but more importantly, the joints, where inadequate treatment may lead to progressive and potentially disabling damage. The case emphasizes the importance of an individualized therapeutic strategy that carefully balances physical and psychological disease burden across different domains.

The main limitations of this study include the small sample size, which restricts the generalizability of our findings, and the absence of biologic-naïve PsA patients, limiting the assessment of baricitinib as a first-line treatment in this population. Additionally, all patients had already achieved adequate control of their psoriatic disease with prior therapies before transitioning to baricitinib, making it difficult to evaluate its efficacy on active, uncontrolled psoriasis. Moreover, no patients showed a particularly extensive cutaneous involvement. This reduces the ability to assess the impact of the drug on severe plaque psoriasis.

## 5. Conclusions

Our experience, although limited to a small group of individuals, highlights the importance of treating multiple concomitant diseases with a single drug in order to minimize the onset of side effects and increase the quality of life of affected patients. Larger and prospective studies including biologic-naïve patients and those with more extensive skin involvement are needed to better define the role of baricitinib in psoriasis and PsA management.

## Figures and Tables

**Table 1:** Demographic and Clinical Characteristics of the Study Population.

Characteristic	Value
Total number of patients	5
Sex	
- Males	2
- Females	3
Age (years)	
- Mean ± SD	53.2 ± 14.3
- Range	27–63
Type of alopecia	
- Alopecia universalis	3
- Severe alopecia areata	2
Patients with psoriatic arthritis (PsA)	3
Clinical forms of cutaneous psoriasis	
- Plaque psoriasis	4
- Guttate psoriasis	1
Reported comorbidities	Arterial hypertension, type II diabetes mellitus, hypothyroidism

## Data Availability

All data generated or analyzed during this study are included in this article. Further enquiries can be directed to the corresponding author.

## Abbreviation

The following acronyms are used in this manuscript:

AA	Alopecia Areata
AU	Alopecia Universalis
JAKi	Janus Kinase Inhibitors
PsA	Psoriatic Arthritis
STAT	Signal Transducer and Activator of Transcription
SALT	Severity of Alopecia Tool
PASI	Psoriasis Area Severity Index
pVAS	Pain Visual Analogue Scale
DMARDs	Disease-Modifying Anti-Rheumatic Drugs
